# Chambers

**Published:** 1866-04

**Authors:** 


### CHAMBERS.

One of the most general, and, at the same time, one of the most pernicious errors in modern architecture, especially in the construction of private dwellings, is founded on the mischievous supposition that almost any place is good enough to sleep in. It is common everywhere to set apart the smallest rooms in the house for sleeping-apartments. To show what a ruinous mistake this is, let the reader remember that at least one third of a man's existence is spent in bed, in sleep. Eight hours out of every twenty-four we are in our chambers. And when it is considered that air is essential to health, that without it we can not live two minutes, it must be of material importance whether we breathe a pure or an impure air for a third of our existence. A full-sized man breathes, takes into his lungs at each breath, about a pint of air; while in there, all the life-nutriment is extracted from it; and, on its being sent out of the body, it is so entirely destitute of life-giving power, that if rebreathed into the lungs again, without the admixture of any pure air, the individual would suffocate, would die in sixty seconds. As a man breathes about eighteen times in a minute, and a pint at each breath, he consumes over two hogsheads of air every hour, or about sixteen hogsheads during the eight hours of sleep; that is, if a man were put in a room which would hold sixteen hogsheads of air, he would, during eight hours sleep, extract from it every atom of life-nutriment, and would die at the end of the eight hours, even if each breath could be kept to itself, provided no air came into the room from without. But when it is remembered, that however pure the air of the whole room was at first, it becomes contaminated by the first expiration, hence only the first, inspiration is pure, and each one thereafter becomes more and more impure, unless there is some ventilating process going on.

Every individual has, in his own experience, demonstrative proof of the impurity of the air of a room in which a person has slept all night, by the "closeness" he has observed on entering a sleeping-apartment after a morning's walk, and this, even when more or less fresh air has been coming in through the crevices about the doors and windows during the whole night. The most eminent physiologists at home and abroad have estimated that no sleeping-apartment, even for a single person, should have a floor surface of less than what would equal twelve feet long and twelve feet broad, or one hundred and forty-four square feet, and eight or ten feet high, or about twelve or fifteen hundred cubic feet to each sleeper. But the sleeping-apartments of hotels, the "state-rooms" of ships, steamboats, and steamships, do not average one third of that cubic space to each sleeper. The state-room of a steamer is ordinarily eight feet long, seven broad, and seven high, and even these are adapted for two sleepers!

As, therefore, each out-breathing vitiates the whole air of a room, as a drop of ink will discolor the whole bulk of water in a tumbler, the chambers for the members of farmers' families should not only be large and commodious, but should be so arranged that a system of ventilation, at least to a small extent, shall be going on all the time, not only in spite of inattention, but a system which can not be easily prevented, which is accomplished by the simple expedient of having a fireplace in each room, which can not be closed with screens or "summer-blowers," for by this means a draft will be made by the cold air coming in at the bottom of the doors and from other places, passing over the floor toward the open fireplace, driving the heavy carbonic acid gas before it up the chimney.

If a neglect of these things were invariably followed by death before morning, attention to them would be compelled. But although the deleterious effects do not thus speedily and impressively follow, they do inevitably result to all persons, under all circumstances—coming on slowly, it is true, but none the less surely and disastrously. To show what a little taint in the atmosphere, not natural to it, may affect the whole system, it is only necessary to state an observed fact, that a man who sleeps near a poppy-field, with the wind blowing toward him from the field, will die before the morning. A canary bird, in its cage, hung to the ceiling of a curtained bed where there were two sleepers, was found dead in the morning. Prof. Carpenter, the first physiologist in Great Britain, ascertained that an atmosphere containing six per cent of carbonic acid gas would produce immediate death, and that less than half that amount would prove fatal in a short time. But every expiration of a sleeper brings out with it some portion of carbonic acid gas, and disperses it through the room; and if six per cent of carbonic acid gas will cause speedy death, the effects of breathing it nightly, even in very small quantities, for twenty or thirty years, can not be otherwise than pernicious to the whole system, must lower the standard of human health, and materially shorten life. But not only is the air in a close room thus constantly being impregnated with carbonic acid gas to the amount of about twenty-eight cubic inches per minute, for each adult sleeper, but the lungs and pores of the skin are constantly discharging an equal amount by weight, that is, three and a half pounds in twenty-four hours, of effete, decaying animal substance, in the form of invisible vapor, which we often see condensed in drops upon the window-glass of crowded rooms, rail-cars, and other vehicles. These drops, if collected and evaporated, have been found to leave a thick putrid mass of animal matter, which is believed to be quite as injurious as carbonic acid gas, if breathed into the lungs; but if not at all injurious, the idea must be abhorrent to every feeling of purity, of taking such a substance into our bodies and incorporating it into the very blood, which is, at the next instant, to be dashed to the lips and tongue for food and nutriment.

In the winter of 1860, a man named Robertson, his wife, and three children, were in the habit of sleeping in one small, ill-ventilated room. One morning, about five o'clock, the wife woke in a very exhausted state, and found her infant of nine months dead in her arms; she immediately aroused her husband, who had barely strength enough to get out of bed; they next discovered that their son of three years of age was also dead, and a daughter of nine in an apparently dying condition, but recovered on being removed to another apartment. Facts like these show that breathing a bad air for a single night is perilous to life, and ought to have an impressive effect on the mind of every man who has a family when he is contemplating building or arranging for them a home for life.

Every chamber, then, should be arranged to have a ventilating process going on all the time, at least by having an open fire-place in it; and as there can be no advantage, but a positive injury, resulting from sleeping in any room colder than forty degrees above zero of Fahrenheit, a little fire should be kept burning in the grate or fire-place, under such circumstances; this creates a draft up the chimney, and keeps the atmosphere of a sleeping-room comparatively pure. In cases where an actual fire can not be kept, an admirable substitute will be found in placing a large lamp in the fire-place, to be kept burning all night; this creates a draft without making much heat, and is a good means of ventilating a sick-chamber when warmth is not desirable, such, for example, as in measles, scarlet fever, and other skin diseases, where a cool air, and at the same time a pure one, is an indispensable means of a safe and speedy cure. But let it be always borne in mind that cold air is not necessarily pure, nor is warm air necessarily impure. With a little fire in a cold bed-room not only is the chamber kept ventilated, but fewer bed-clothes are needed, less clothing does more good next day, while there is a freer escape of gases and exhalations from the body of the sleeper, and the person wakes up in the morning more fresh and vigorous.

Chambers should not only be constructed with a view to a constant, thorough, and unpreventable ventilation, but also with an eye to their perfect dryness and their free exposure to the sun for the greater portion of every day. Florence Nightingale, that beautiful name and more beautiful character, which will go down to posterity with that of John Howard and Dorothea Dix, and others of nature's nobility, writes after long years of experience with the sick and suffering :

" A dark house is always an unhealthy house, always an ill-aired house, always a dirty house. Want of light stops growth, and promotes scrofula, rickets, etc., among children. People lose their health in a dark house, and if they get ill they can not get well again in it. Three out of many negligences and ignorances in managing the health of houses generally, I will here mention as specimens. First, that the female head in charge of any building does not think it necessary to visit every hole and corner of it every day. How can she expect that those under her will be more careful to maintain her house in a healthy condition than she who is in charge of it? Second, that it is not considered essential to air, to sun and clean rooms while uninhabited; which is simply ignoring the first elementary notion of sanitary things, and laying the ground for all kinds of diseases. Third, that one window is considered enough to air a room. Don't imagine that if you who are in charge don't look to all these things yourself, those under you will be more careful than you are. It appears as if the part of the mistress was to complain of her servants and to accept their excuses—not to show them how there need be neither complaints nor excuses made."

In reference to the same subject, and in confirmation of what has been already stated in this article, Dr. Moore, the metaphysician, thus speaks of the effect of light on body and mind: " A tad-pole confined in darkness would never become a frog; and an infant being deprived of heaven's free light will only grow into a shapeless idiot, instead of a beautiful and responsible being. Hence, in the deep, dark gorges and ravines of the Swiss Valais, where the direct sunshine never reaches, the hideous prevalence of idiocy startles the traveler. It is a strange, melancholy idiocy. Many citizens are incapable of any articulate speech; some are deaf, some are blind, some labor under all these privations, and all are misshapen in almost every part of the body. I believe there is in all places a marked difference in the healthiness of houses according to their aspect with regard to the sun, and those are decidedly the healthiest, other things being equal, in which all the rooms are, during some part of the day, fully exposed to the direct light. Epidemics attack inhabitants on the shady side of the street, and totally exempt those on the other side; and even in epidemics, such as ague, the morbid influence is often thus partial in its labors."

